# *Pinus halepensis* in Contaminated Mining Sites: Study of the Transfer of Metals in the Plant–Soil System Using the BCR Procedure

**DOI:** 10.3390/toxics10120728

**Published:** 2022-11-26

**Authors:** Pegah Kharazian, Emilia Fernández-Ondoño, María Noelia Jiménez, Manuel Sierra Aragón, Antonio Aguirre-Arcos, Gianluigi Bacchetta, Giovanna Cappai, Giovanni De Giudici

**Affiliations:** 1 Department of Chemical and Geological Sciences, University of Cagliari, Cittadella Universitaria di Monserrato-Blocco A, 09042 Monserrato CA, Italy; 2 Department of Soil Science and Agricultural Chemistry, Faculty of Science, University of Granada, C/Severo Ochoa, s/n, 18071 Granada, Spain; 3 Department of Botany, Faculty of Pharmacy, Campus de Cartuja s/n, University of Granada, 18071 Granada, Spain; 4 Department of Life and Environmental Sciences, Centre for the Conservation of Biodiversity (CCB), University of Cagliari, Viale Sant’Ignazio da Laconi 11-13, 09123 Cagliari, Italy; 5 Department of Civil-Environmental Engineering and Architecture, University of Cagliari, Piazza d’Armi 1, 09123 Cagliari, Italy

**Keywords:** mine tailing, BCR sequential extraction, phytoremediation, phytostabilization, *Pinus halepensis*, geochemical characteristics

## Abstract

The study aimed at evaluating the geochemical fractions of Zn, Pb, Cd and their bioavailability in soil in-depth and around the root of *Pinus halepensis* grown on heavily contaminated mine tailing in south-western Sardinia, Italy. The contaminated substrates were partly investigated in a previous study and are composed of pyrite, dolomite, calcite, quartz, gypsum, barite, iron-sulfate and iron-oxide. The geochemical fractions and bioavailability of Zn, Pb and Cd were measured through the BCR extractions method. Cadmium in the superficial contaminated substrates was mainly found in the exchangeable BCR fraction. Zinc and lead were often found in the residual BCR fraction. PCA confirmed that the uppermost alkaline-calcareous layers of mine waste were different with respect to the deeper acidic layers. We demonstrated that Pb and Zn were less present in the exchangeable form around the roots of *P. halepensis* and in soil depth. This can be due to uptake or other beneficial effect of rhizospheres interaction processes. Further studies will shed light to confirm if *P. halepensis* is a good candidate to apply phytostabilization in mine tailing.

## 1. Introduction

Mine wastes are among the most hazardous sources of metal contamination for the surrounding area. Most often, minerals in mine tailing sites undergo oxidation and dissolution due to exposure to atmospheric agents [1]. Indeed, mine tailings are pollution sources often subjected to the mobilization and dispersion of metal contaminants by wind, seasonal heavy rainfalls, water run-off and water erosion [2,3] and can seriously spread a high range of trace element contaminants in soils, ground-waters and plants [4,5].

Some plant species that have adapted to grow in highly contaminated environments have been suggested as some of the most feasible and effective tools in phytostabilization through bio-mineralization processes [6,7]. This can eventually play an important role in the immobilization of elements around the root systems and the recovery of the polluted mine sites [8,9,10]. In mine waste environments, soil elements can be associated with different geochemical forms, such as soluble exchangeable, carbonates, iron-manganese oxides, residues and organic materials. Indeed, the elements with the highest bioavailable potential are mainly those related to the water-soluble and exchangeable fraction in the soil–plant system, whereas the residual fraction is considered not to be bioavailable for plants [11]. The sequential extraction method is generally used for assessing the potential of element mobility in different geochemical forms and analyzing their bioavailability [12,13,14]. The BCR three-step sequential extraction method (hereafter BCR) is an analytical tool that has been proposed by the European Community Bureau of References [13,15] and is widely used for performing element extraction analysis in mines. In this method, metals are extracted using chemical reagents in three steps and four fractions. In fact, the BCR method detects the different metals in the forms of (i) weak acid-soluble or exchangeable fraction bound to carbonates, (ii) reducible fraction related to Fe and Mn oxides, (iii) oxidizable fraction (organic matter and sulfides), and (iv) residual fraction bound to silicate and mineral structures that is a relatively resistant and stable fraction [16,17,18].

The hypothesis of this work is that the geochemical fractions of Cd, Zn and Pb can be controlled by the physico-chemical properties and the mineralogical composition of soil as well as the plant root system. On one side, the physico-chemical properties of soil, such as pH, electrical conductivity, organic carbon, soil texture and the total metal concentration of elements in soil can affect the solubility of elements and the bioavailable fractions in soil [19,20,21], waste and sediments [22,23]. On the other hand, the mineralogical characteristics of soil [2] and the plant root activity [9] can rule trace element bioavailability. Hence, it is important to assess the mobility of elements and specify the stability of different forms of elements considering both the mineralogical and physico-chemical characteristics in the soil–plant system.

In this study, the soil and plants were from south-west Sardinia, an Italian mine area with a rich history of mine activities that have left large quantities of mine wastes in dumps and flotation tailings. The environmental risk of the area has increased due to inadequate actions for the mitigation of metal impact after the mines shut down [24]. Several pilot projects have been carried out focused on the rich plant diversity of the areas in order to identify the most suitable autochthonous plant species possessing the best phytoremediation capabilities to be exploited [25,26,27].

*Pinus halepensis* Mill. is a Mediterranean widespread tree species that has been frequently noted for its ability to tolerate high amounts of metals [9,28] and for the restoration of degraded soil in arid and semi-arid areas [29,30], as well as the degraded and low fertile soils of mine wastes [31]. To our knowledge, little attention has been devoted to the study of the geochemical fractions of elements in the soil–root system of *P. halepensis* as well as in the different depth layers of soil where it grows spontaneously in the multiple heavy metal-contaminated mine tailing.

A preliminary study was carried out in the abandoned mine tailing site of Campo Pisano (SW-Sardinia) by our research group in 2020 to investigate the contaminated soils and different compartments (roots, barks, wood and needles) of *Pinus halepensis* and to evaluate the metal content, plant accumulation and translocation behavior as well as the main mineralogical characteristics of Zn, Pb and Cd [32]. The findings showed that despite the detection of a high concentration of metals in the soil, the estimated Biological Accumulation Coefficient and Translocation Factor (BAC, BCF and TF < 1) were very low. These findings indicated that *P. halepensis* has a qualified metal toleration capability and has limited metal accumulation and translocation factors in the aerial parts. Thus, this plant can be considered as an excluder plant and a candidate for phytostabilization projects [32].

The aim of this study was to determine the geochemical fractions of Zn, Pb and Cd and the specific geochemical forms of elements present in the same sampling site through the three-step BCR extraction method in relation to the physico-chemical and mineralogical characteristics of the soil in depth and the soil–root system. In order to pursue this aim, the study exploited the pertinent data of mineralogical and chemical analysis of contaminated soil and root samples available in Kharazian et al. (2022) [32].

## 2. Materials and Methods

### 2.1. Study Area

**Figure 1:**
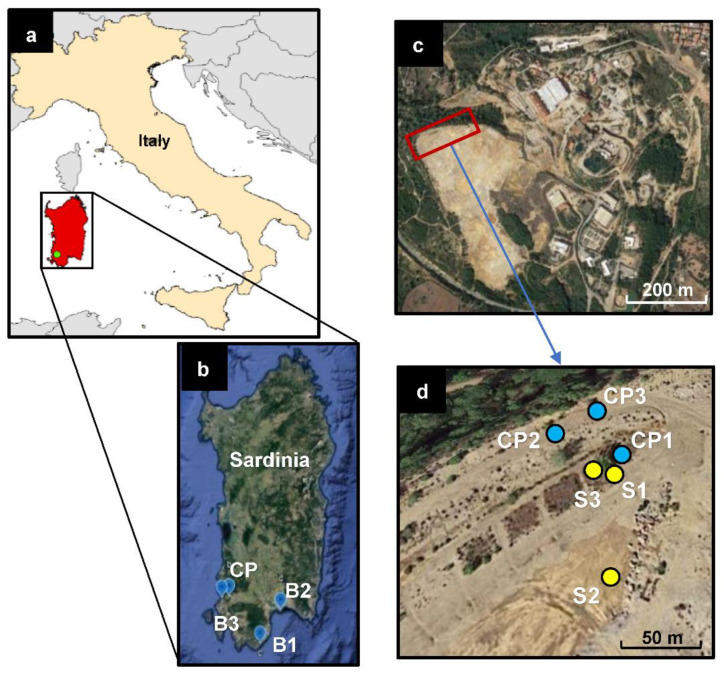
Location map of Sardinia (**a**); sampling sites of the collected soils around the root of *Pinus halepensis* in the contaminated Campo Pisano site (CP) and not-contaminated sites (Santa Margherita: B1; Calamosca: B2; and Fontanamare: B3) (**b**); location map of the study area (**c**); details of the sampling points and soil samples collected in the CP-contaminated site: the core-drilled soil samples are indicated by yellow spots (S1, S2 and S3); and the blue spots indicate the soil around the root in contaminated (CP1, CP2 and CP3) and non-contaminated sites (B1, B2 and B3) (**d**).

The study area was Campo Pisano (CP) mine tailings dump that belongs to the Metalliferous Ring of the Sulcis-Iglesiente mining district, one of the most important mine regions of Europe since pre-Roman times, located in south-western Sardinia, Italy (Figure 1). The area has a Mediterranean pluviseasonal bioclimate with upper thermo-Mediterranean thermotypes and ombrotypes between the lower sub-humid and the upper dry [33]. The Campo Pisano ore area belongs to the Metalliferous Ring of Sulcis-Iglesiente mining district and geologically is characterized as a Paleozoic carbonate with the middle Cambrian limestone rocks and pre-Variscan sulfides (Zn and Pb) and non-sulfide deposits [34]. The main common minerals are pyrite (FeS_2_) cerussite (PbCO_3_) and anglesite (PbSO_4_), associated with nodules and residual galena (PbS), dolomite (CaMg (CO_3_)_2_), calcite (CaCO_3_), quartz (SiO_2_), barite (BaSO_4_) and iron hydroxides [35]. The CP mine was extensively exploited before it became inactive in 1998 [36]. The mine also exploited pyritic ores from Genna Luas, leading to tailings rich in pyrite. It should be noted that the surface layer of the Campo Pisano area is characterized by a carbonate lithology rich in alkaline waste materials coming from the Monteponi area (SW-Sardinia) [5,34]. This alkaline cover was applied to avoid the direct contact of air and water with the acid and pyrite-rich tailings. Moreover, the metal concentrations of CP mine wastes are highly heterogeneous due to the different extraction methods applied for mine exploitation activities [25,36].

In this study, the term soil refers to the collected mine waste samples. In the previous research study, between the years 2008 and 2010, a successful phytoremediation study on some plant species was carried out in an experimental plot amended with compost produced from the organic fraction of Municipal Solid Waste (MSW) in - CP tailing site [10,37]. The contaminated sampling points of this study are located in the same experimental site and its surrounding area.

### 2.2. Sampling

Six *Pinus halepensis* specimens with a nearly similar height (2 m) and age (10–12 years old) were selected from both non-contaminated and contaminated sites in the south-west of Sardinia (November 2020) where they grow spontaneously (Figure 1a,b). Six bulk soil samples were collected from the soils around the roots of *P. halepensis* together with its root samples: (i) CP1: located inside the contaminated Campo Pisano (CP) site in the compost-amended plot aged 10 years old [10,37], (ii) CP2: outside of the CP experimental plot distancing 3–4 m from it aged 10 years old, and (iii) CP3: located outside of the CP experimental plot distanced 6–7 m from it and aged 12 years old (Figure 1c,d) (see more in [32]), (iv) B1: Blank sample in the non-contaminated site (Santa Margherita, Pula) at approximately 80 km far away from CP mine site, (v) B2: Blank sample in the non-contaminated site (Calamosca, Cagliari) at about 60 km far away from the CP mine site, (vi) B3: Blank sample from the less-contaminated site (Fontanamare, Gonnesa) at about 10 km distance from the CP mine site (Figure 1b). The contaminated soils around the root and root samples were exploited the previous study [32].

Three soil in-depth core-drilled samples (approximately 70 cm) were also collected from the CP-contaminated mine sites using a core sampler (Atlas Copco’s COBRA): (i) the first sample in the experimental plot at approximately 50 cm distance from the tree (S1), (ii) the second one in a bare site, out of the experimental plot, where there is no vegetation canopy (S2), (iii) the third sample in the amended plot at 1–2 m distance from the pine (S3) (Figure 1d); see more in [32]. The three soil samples were selected from each core-drilled sample, according to the visually recognizable color of the soil substrate and their mineralogical and geochemical characteristics that were investigated in our previous research study [32]. These layers were selected for BCR analysis, regardless of their soil depth horizon, to provide a better interpretation of their metal availability. Samples were named as: uppermost soil [**1** (0–20 cm): S1-1, S2-1, S3-1)], interval depth [**2**: S1-2 (20–28 cm), S2-2 (37–41 cm), S3-2 (20–45 cm)], and subsamples [**3**: S1-3 (32–38 cm), S2-3 (47–50 cm), S3-3 (56–66 cm)]. The 20 cm uppermost soil of each collected core-drilled soil was considered the uppermost soil sample. Figure 1c,d provides the CP site location where the core-drilled soil samples were collected (see more about the collected samples in Supplementary Materials, Table SM1 and Figure SM2).

### 2.3. Physico-Chemical and Mineralogical Characterizations of the Soil and Root

Samples, including bulk soils (rhizospheres solid materials), the soil grain particles around the plant roots (within 3–5 mm around the root) and the soil in deep layers as well as root samples were all air-dried (for almost a week) at room temperature after gentle shaking, wiping and removal of the remaining particles. A detailed description of sample preparation and the chemical and mineralogical characteristics of the contaminated soils and root samples can be found in Kharazian et al. (2022) [32]. Sample preparation and both SEM and XRD analyses were carried out at the Center for Research University Services (CESAR), Cagliari University, Italy, and the remaining chemical and physical analyses, BCR sequential extractions, DTPA extractions and ICP-OES analyses were performed at the Department of Edaphology and Agricultural Chemistry, University of Granada, Spain.

#### 2.3.1. Physical and Chemical Properties of the Soil

Soil pH was measured in 25 mL distilled water with 10 g soil (ratio of 1:2.5) using a conductometer/pH-meter (914 Metrohm). Electrical Conductivity (EC) of the soil was obtained in distilled water (1:1) utilizing a conductivity meter (Eutech CON700). Calcium carbonate content (equivalent CaCO_3_) was calculated following the method proposed by the Soil Conservation Service (1972) [38]. Total carbon and nitrogen as well as organic carbon (OC) contents of the substrates were determined through an elemental analyzer LECO ^®^ (TruSpec CN, St. Joseph, MI, USA) calibrated with the reference material (ore minerals). Organic carbon (OC) was analyzed after the soil samples were treated and acid-washed (HCl 1 mol L^−1^) to remove carbonate content, according to Ussiri and Lal (2008) [39]. Amorphous forms of iron and aluminum (Fe and Al oxide) were extracted with 1 M ammonium oxalate and oxalic acid following the Schwertmann and Taylor (1977) procedure and measured by the Inductively Coupled Plasma Optical Emission in a Spectrometer ICP-OES (Perkin Elmer Avio ^®^ 500, Waltham, MA, USA) [40].

#### 2.3.2. Mineralogical Characterization of the Soil and Root

Soil and root samples were analyzed through XRD analysis using laboratory equipment (Pan analytical X’Pert Pro, X’Celerator detector) and software X’Pert High Score Plus (PAN-analytical B.V., Almelo, The Netherlands) to clarify the mineral phases. The microscopic characteristics, as well as the element distribution of samples, were clarified under low-pressure conditions through Energy Dispersive Spectroscopy (Thermo Ultra Dry EDS Detector, Pathfinder, Waltham, MA, USA) and Scanning Electron Microscopy (SEM) imaging (ESEM QUANTA 200, FEI) [26]. The detailed description and the outcome of the mineralogical characteristics (XRD and SEM) of the CP-contaminated soil and root samples can be found in Kharazian et al. (2022) [32].

#### 2.3.3. Total Metal Concentration of the Soil and Root

The chemical characteristics of soil samples were analyzed following the official Italian analytical methods (D.M. 13-09-1999) [41,42] and the total metal concentrations in soil and root samples were assessed using the Environmental Protection Agency (EPA) method 3052 [24]. Total metal concentration in the soil was assessed using acid digestion (9 mL concentrated HNO_3_ 65% and 3ml of HF 48%) through the laboratory microwave system (CEM Mars^®^ XP1500 Plus, Mathews, CN, USA). Zn, Pb and Cd concentrations of both root and soil samples were determined by ICP-OES, Perkin Elmer Avio^®^ 500, Waltham, MA, USA. The detailed description of the method performed was reported in Kharazian et al. (2022) [32]. We also calculated the Biological Concentration Factor (BCF) as the ratio between the metal content in the roots and in soil samples that are available in [32].

#### 2.3.4. Bioavailable Concentration of Elements (DTPA)

The bioavailable contents of Zn, Pb and Cd were evaluated for all contaminated and non-contaminated soils around the roots through a single extract method performed by using 0.005 M DTPA solution (1.96 g of DTPA, 14.92 g triethanolamine and 1.47 g of CaCl_2_.2H_2_O dissolved in the final solution of 1L by distilled water adjusted to pH~7.3) added to the substrate samples [41,43]. The concentration of the extracted metal was measured through the ICP-OES technique (the Agilent 725-ES method). DTPA extraction is considered as an efficient procedure that prevents the carbonate’s dissolution and consequently, the release of the bounded metals [42,44]. The metal bioavailability in soil was calculated in terms of the percentage values according to Equation (1).

DTPA bioavailability (%) = (M_Metal bioavailability in soil_)/(M_Total metal concentration in soil_) × 100 (1)

#### 2.3.5. Sequential Extraction Procedure (BCR)

The three-step BCR sequential extraction method of the European Community Bureau of Reference was applied to differentiate metal fractions in all collected soils [13,45]. Accordingly, the four extracted fractions were defined as exchangeable (F1); reducible (F2); oxidizable (F3) and residual (F4) fractions performed through the application of modified reagents in 1 g soil. The mixture of each fraction was centrifuged for separating and measuring the extracted metals through the ICP-OES technique. The remaining materials at the end of each BCR fraction were washed with 20 mL distilled water, shaken for half an hour and centrifuged to be prepared for the next fraction step.

The exchangeable fraction (F1) was performed by extracting metals during 16 h shaking and using 40 mL of 0.11 mol/L acetic acid. In the reducible fraction (F2), 40 mL of 0.5 mol/L hydroxylamine hydrochloride at pH 2.0 was added to the residue of the first step and shaken for 16 h. In the oxidizable fraction (F3), 50 mL of 1 M ammonium acetate (adjusted at pH = 2 with HNO_3_) was applied following the oxidation process with 10 mL of H_2_O_2_ (acid-adjusted pH 2) for an hour at room temperature and an hour heated to 85 °C with occasional agitation (PerkinElmer-SPB 50-48S). The same process was repeated until the liquid volume was <1 mL. The residual remained material in the fourth extraction (F4) was oven-dried at 45 °C for a day and acid digested with the same digestion procedure as the total metal concentrations. The Zn, Pb and Cd concentration extracted in each BCR fraction was determined through Inductively Coupled Plasma Optical Emission in a Spectrometer (ICP-OES, Perkin Elmer Avio^®^ 500, Waltham, MA, USA).

The precision of the chemical test was evaluated by performing a triplicate sample. Blank solutions and different reference materials were applied to ensure the reliability of the analytical methods in the analysis of the total metal content of the soil samples. The analytical quality control and the accuracy of the analyzed data were verified by analyzing a certified reference material for soil (CRM052-050, Sigma-Aldrich, USA, St. Louis, MO, USA). All standards and blanks were matrix-matched with the samples and reagents.

The ratios between the metal concentrations (mg kg^−1^) of each BCR fraction (F1, F2, F3 and F4) and the total metal concentration in all BCR fractions were calculated (in percent) for all samples. Moreover, to confirm the reliability of the BCR outcomes, the ratios between the sum of BCR fractions (F1, F2, F3 and F4) and the total metal concentration for each element were measured in all soil samples. The sum of the first three extractions represents the available fraction and correlates with soil properties, such as elements adsorbed onto mineral surfaces, reactivity and solubility of minerals [23]. The values generally corresponded to the recovery (in percent) of the BCR extraction method and varied from 70.1% to 126.9 %. The variable and poor recovery calculated for some elements could be described by the high heterogeneity characteristics of mine waste samples [46,47] and the process of washing samples with distilled water at the end of each BCR fraction step [48].

### 2.4. Data Analysis

Statistical analyses were performed using IBM SPSS Statistics 23.0. The result values are expressed as the mean ± standard deviation and the significant level of statistical analysis in all cases considered <0.05. The correlation among metal extracted in BCR fractions with total metal contents in soils, metal concentrations in roots, soil properties and the concentration of DTPA extracted metals were explored using Pearson correlations coefficients. The matrix of Principal Component Analysis (PCA) was carried out to examine the correlations between measured parameters of elements in BCR fractions, all physico-chemical soil properties and DTPA-extracted metals in the contaminated and non-contaminated soil samples using the statistical software CANOCO 5 following the recommendations proposed by ter Braak and Smilauer (2002) and Lepš and Šmilauer (2003) [49,50].

## 3. Results

### 3.1. Physical and Chemical Soil Properties

Table 1 summarizes the physico-chemical properties of all the examined soil samples. The data show that the pH values of CP soil samples around the *Pinus halepensis* root varied from 7.4 (CP3) and 7.7 (CP2), whereas the in-depth soil samples were generally more acidic than the upper ones. Indeed, the pH showed a higher value in the uppermost soil layer of the amended plot samples S1-1 (6.8) and S3-1 (6.9) than in the soil depth S1-3 (2.3) and S3-3 (2.5). The carbonate content of soil samples was high in all CP soils around the roots (49.7% in CP1, 45.4% in CP2 and 41.03% in CP3). Moreover, the highest value was detected in the uppermost soil samples: S2 (59.4%) > S3 (53.6%) > S1 (52.07%), while it decreased in the deep layer of the amended plot with the lowest (0.19%) in the amended deep layer S1-3 (Table 1). The values of total carbon (TC) and nitrogen (TN) content of the CP soil around the *P. halepensis* root ranged from 5.65% to 9.97% and from 0.27% to 0.70%, respectively. In the deeper soil layers, the TC and TN contents of the soils were decreased in the amended plots S1 (0.36% TC, 0.03% TN) and S3 (0.38% TC, 0.02% TN).

The highest organic carbon (OC) content was measured in the uppermost soil layer of the compost-amended plot sample S1-1 (1.9%), whereas the lowest was in the deeper soil layer of S1-3 (0.02%) where Electrical Conductivity (EC) reported its highest value (29.3 dS m^−1^). Table 1 shows that Fe and Al oxide had different concentrations in the deep layers of the soil samples. The lower soil layer of the amended plot S3-3 showed the highest amount of Fe- oxide (up to 42,948.9 mg kg^−1^). Moreover, Al-oxide content was high in the CP soil around the roots and in the upper most soil samples.

### 3.2. Mineral Composition in the Soil and Plant Roots

**Figure 2:**
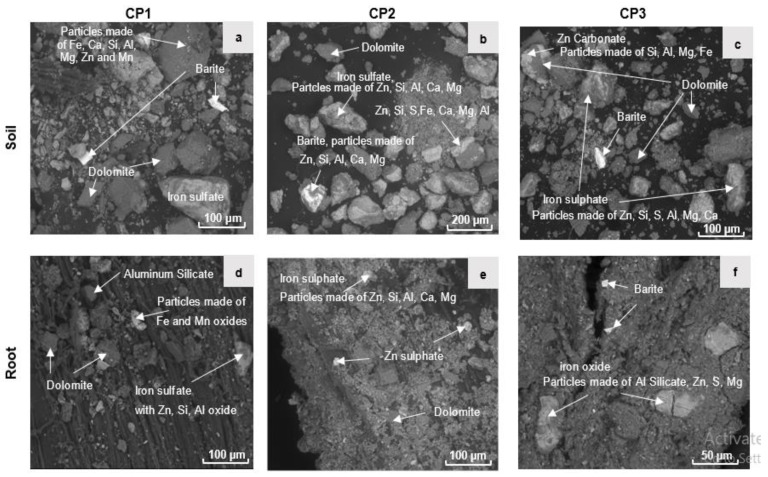
Spectroscopy-scanning Electron Microscopy (SEM) analysis on minerals of the root and the soil around the root of *Pinus halepensis* samples (CP1, CP2 and CP3)*;* in the soil samples (**a**–**c**), and the root surface (**d**–**f**).

The XRD analysis performed on the collected bulk soil samples shows that carbonate (dolomite: CaMg(CO_3_)_2_), silica, quartz (SiO_2_), and gypsum (CaSO_4_) were the predominating minerals in all soil samples; see more in [32]. It shows that iron sulfide (pyrite) was found in all bulk soil layers, except in the layers of the amended plots S1-2 and S3-3. Moreover, SEM analysis detected iron oxide (goethite) and iron-sulfate (jarosite) in all the soil around the root samples (Figure 2a–c) together with zinc carbonate (smithsonite) (Figure 2c). Muscovite (KAl_2_ [(AlSi_3_O_10_) (OH)_2_] was present in the uppermost soil samples of amended plots S1 and S3 (0–20 cm) and the soil around the roots of CP3 (Figure 2c). Moreover, illite [(K, H_3_O) (Al, Mg, Fe)_2_ (Si, Al)_4_ O_10_ (OH)_2_, (H_2_O)] was found in the bulk soil samples S2-2, S3-2 and CP1 and the main minerals detected through XRD analysis on the root samples were dolomite, whewellite (Ca (C_2_O_4_)∙2(H_2_O), silica and barite (Supplementary Materials, Figure SM3). The SEM analysis performed on soils and particle materials shows that these mineral particles were embedded and adhered to the external part of *Pinus halepensis* root samples and mostly detected iron-sulfate (Figure 2d,e) and iron-oxide phases (Figure 2f), as well as a mixture of other elements, mainly Al, Si, Zn and Fe, embedded on the external part of the root samples (Figure 2d–f) (see more in the supplementary materials, Figure SM4).

### 3.3. Total Zn, Pb and Cd Concentrations

**Figure 3:**
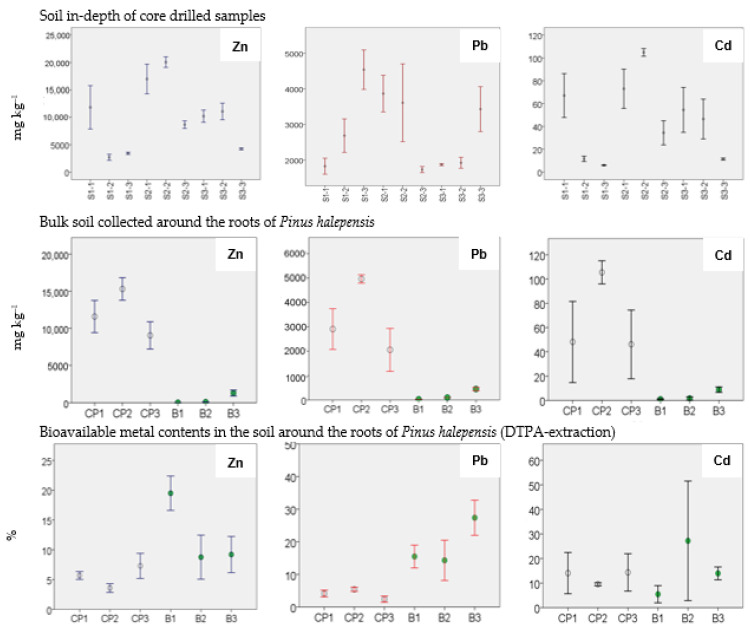
Total Zn, Pb and Cd content (mean values and standard deviation) in core-drilled samples at different depths. Total metal content and bioavailable fraction (DTPA extraction) in soil around the roots of *Pinus halepensis* at Campo Pisano (CP1, CP2 and CP3) and in non-contaminated sites (B1, B2 and B3); non-contaminated soils are indicated with green circles.

The chemical data of Zn, Pb and Cd concentration in all contaminated soil samples showed that Zn was the most abundant metal followed by Pb (Figure 3); see more in [32]. In the non-contaminated soil samples, total metal contents ranged from 10.2 to 1305.7 mg kg^−1^ for Zn, 27.6 to 450.7 mg kg^−1^ for Pb and 0.88 to 8.9 mg kg^−1^ for Cd. In the contaminated soil samples, CP2 had the highest total Zn, Pb and Cd content in the soil around the root samples. Moreover, Zn and Cd concentrations decreased in the lower soil layers of the amended plot S1-3 (3421.5 mg kg^−1^ for Zn and 5.9 mg kg^−1^ for Cd) and S3-3 (4224.8 mg kg^−1^ for Zn and 11.4 mg kg^−1^ for Cd). However, in the same samples, the total content of Pb was higher and ranged from 3430.04 in S3-3 to 4537.6 mg kg^−1^ in S1-3 (Figure 3). The BCF values and the data for the root samples were taken from the previous study available in [32] (see more in the supplementary materials, Figure SM5 and SM6).

### 3.4. Bioavailable Content of Zn, Pb and Cd (DTPA)

Figure 3 reports the bioavailable metal content (percentage) of the soil collected around the roots in CP-contaminated (CP1, CP2 and CP3) and in non-contaminated sites (B1, B2 and B3). The data of contaminated soil around the root sample CP3 showed the highest Cd and Zn bioavailable fraction (13.8% Cd > 7.3% Zn), and the lowest bioavailability of Pb (2.4%); see more in [32]. The values show that the Cd bioavailable fraction was remarkably high in all contaminated soils around the roots from 9.5% in CP2 to 13.8 % in CP3 and 13.5% in CP1. The data show a variable bioavailable fraction in non-contaminated soils with the highest Pb (27.3%) in B3, Cd (25.2%) in B2 and Zn (19.5%) in B1. Thus, this test confirmed that contaminated samples had a significant bioavailable fraction, even below 20% of the total amount. As the bioavailable fraction was much lower than the bulk concentration, these data are relevant for a risk analysis of the Campo Pisano area.

### 3.5. The BCR Sequential Extraction

**Figure 4:**
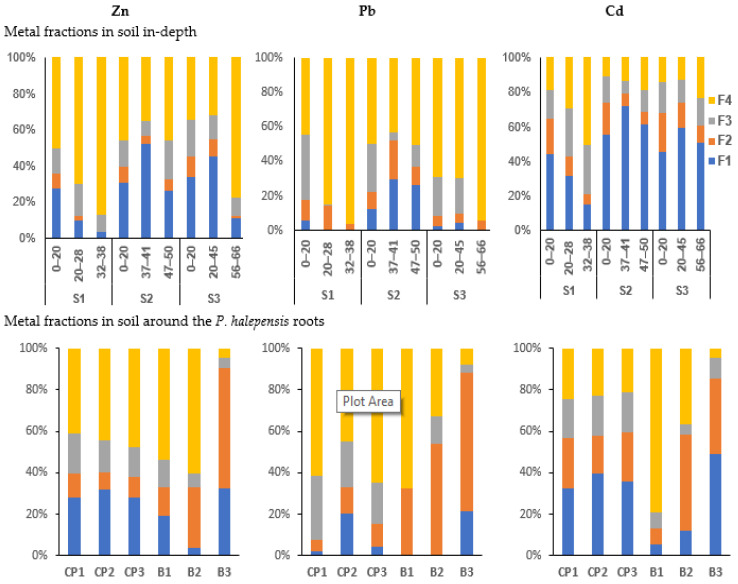
Geochemical fractions of Zn, Pb and Cd obtained through BCR sequential extraction: acid-extractable (F1), reducible (F2), oxidizable (F3) and residual fraction (F4) in the soil collected from depth layers of the core samples (S1, S2 and S3) and around the collected *Pinus halepensis* root samples in Campo Pisano mine tailings (CP1, CP2, and CP3) and in the non-contaminated areas (B1, B2 and B3).

Figure 4 shows the geochemical fractions (percentage) of Zn, Pb and Cd in all soil samples through the BCR sequential extraction method. The data show that in CP soils around the roots and in the uppermost soil layers, Zn and Pb were often present in the residual fraction (F4), whereas the highest fraction of Cd was in the exchangeable fraction (F1). The data show that the highest Pb was present in F4 in the deep layers of the amended plot samples S1-3 (96.2%) and S3-3 (94.1%) where it was less bound to the exchangeable fraction (F1) (Figure 4). Moreover, in all CP soil samples, some part of Zn was mainly present in exchangeable fraction F1 (ranging from 10.8% to 51.8%) more than in the oxidizable fraction F3 (7.9%–22.1%) > F2 (0.1–11.5%). The highest Zn in F1 was reported in S2-2 (51.8%) where it showed the highest total metal content and there was no vegetation.

Unlike CP-contaminated soils, the non-contaminated and less contaminated soils showed variable extracted elements in BCR fractions. For instance, in the less contaminated soil sample (B3), Zn and Pb were more present in F2 (66.8% Pb > 57.9% Zn) and F1 (32.5% Zn > 21.4% Pb) than F4 (7.6% Pb > 4.6% Zn) and F3 (5.1% Zn > 4.1% Pb) (Supplementary Materials, Table SM2).

### 3.6. Correlation between BCR Fractions and Metal Content in the Soil and in the Plant–Root System

The Pearson correlation between Zn, Pb and Cd extracted in the BCR fractions with the total metal content in the soil and in the *Pinus halepensis* root samples, the concentrations of DTPA-extracted metals, the sum of all BCR fractions and the soil properties are shown in Table 2. The soil core-drilled samples and the soil around the roots were considered separately in order to indicate the details of the data correlations. In the soils around the roots, all metals (Zn, Pb and Cd) bound in F1 appeared to be positively correlated with their concentrations in soil and *P. halepensis* root samples. Moreover, the concentrations of Zn and Cd extracted in all BCR fractions were highly positively correlated with their concentration of DTPA-exchangeable availability in the same soil samples. The matrix shows that the total Zn concentration in the soil and Cd concentration in roots samples were significantly positively correlated with Zn extraction in all BCR fractions. The Fe-oxide content in the soil around the roots samples was significantly positively correlated with all elements extracted in the BCR fractions, except for Cd in the residual fraction (F4). In the same soil sample, all metals in F4 appeared to be positively correlated with the CaCO_3_ content and only Zn and Pb showed a positive correlation with Fe-oxide in the same fraction. In the core soil samples, CaCO_3_ presented a positive correlation with Zn and Cd concentration extracted in F2 and F3. The concentration of Cd found in F2 and F3 correlated positively with Al-oxide in the soil core-drilled samples, while there was no reported positive correlation in the soil around the roots for Al-oxide in the BCR steps.

**Figure 5:**
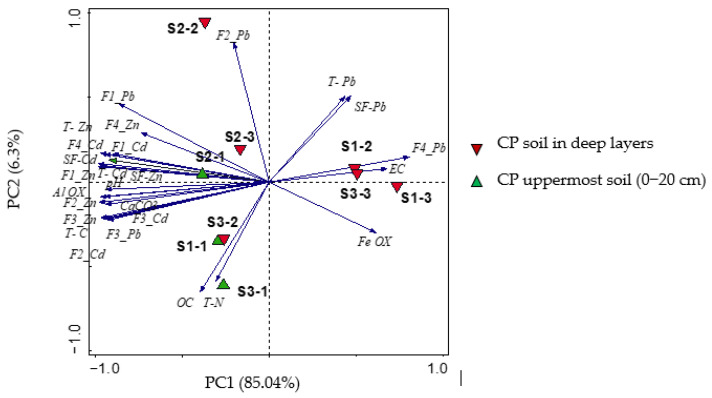
PCA for the collected soils in deep layers. Elements extracted in F1, F2, F3 and F4 BCR Fractions; the total metal content in soil (T-Zn; T-Pb; T-Cd); the sum of the BCR fractions (SF-Zn; SF-Pb; SF-Cd); electrical conductivity (EC); organic carbon (OC); total nitrogen (T-N); total carbon (T-C) in the uppermost soil samples (S1-1, S2-1 and S3-1) and the deep layers (S1-2, S1-3, S2-2, S2-3, S3-2, S3-3).

The PCA was performed to assess the distribution and similarity among the collected soil samples and the correlation between Zn, Pb and Cd extracted in all standardized BCR fractions with soil properties and total metal concentration in the soil and root samples. The parameters were spatially ordinated within a diagram according to the collected soil in deep layers (S1, S2 and S3) (Figure 5) and the soils around the root of the *P. halepensis* samples on the non-contaminated soil (B1, B2 and B3) and contaminated CP soils (CP1, CP2 and CP3) (see more in Supplementary Materials, Figure SM7).

Figure 5 shows that the first component of the diagram (PC1) had 85.04% of the total variation. Different parameters, such as Fe, EC, residual Pb (F4-Pb), total Pb (T-Pb) and the sum of BCR for Pb (FS-Pb) were all presented in PC1 with a positive correlation. The other parameters, such as CaCO_3_, pH, Al-oxide as well as Zn and Cd extracted in F2 and F3 showed strongly negative correlations in the inverse section of the first principal component. The second principal component (PC2) explained 6.3 % of the total variation with a highly positive correlation for Pb extracted in the second fraction (F2-Pb) and a negative correlation for organic carbon (OC) and total nitrogen (T-N) on PC2. The deep layers of the soil-amended plot samples S1-2, S1-3 and S3-3 are distributed on the positive side of the first axis and the main variables that had a positive correlation with them were Fe-oxide, EC, the total content of Pb (T-Pb), residual of Pb (F4-Pb) and the sum of BCR fractions for Pb (SF-Pb). Moreover, the uppermost soil samples S1-1, S2-1 and S3-1 are distributed on the negative side of the first component axis and exhibited high levels of CaCO_3_, pH, Zn and Cd extracted in all BCR fractions and the sum of BCR fractions. Further, the availability of Zn and Cd extracted through the BCR fractions decreased in the deep soil layers of the amended plots S1-3 and S3-3, which had high EC, Fe-oxide and residual Pb (Figure 5).

## 4. Discussion

The CP soil samples had a high content of Zn, Pb and Cd due to the high concentration of heavy metals originated from the minerals present in the extremely polluted mine site [51]. On the other hand, the heterogeneous mineral composition and the different physico-chemical properties of CP soils could affect the mobility of elements in soil and root samples. Thus, the BCR fractions were evaluated in relation with the soil properties and physico-chemical and mineralogical characteristics of soil in depth and around the root samples.

### 4.1. The Mineralogical Investigation Related to the Soil Properties

The mineralogy of the investigated soils comprised primary minerals, i.e., metal sulfide (pyrite), carbonate (calcite and dolomite) and quartz as well as the secondary mineral, i.e., iron-sulfate (jarosite) and iron hydroxides, in agreement with previous studies carried out by De Giudici et al. (2015) and Lai et al. (2015) [8,10]. In fact, the formation of secondary minerals compounds is generally due to the weathering minerals process or induced by biogeochemical processes occurring in the soil–root system of the plant. Sulfate minerals, such as jarosite (iron sulfate) and gypsum (Calcium sulfate) are formed during the pyrite (iron sulfide) oxidative–dissolution process [52,53]. This can increase the dissolved ions and eventually elevate the electrical conductivity (EC) in S1-3 and S3-3. PCA showed that the surface layer was different with respect to the deep layer. Surface layers showed alkaline pH values consistent with alkaline and carbonate lithology of the Monteponi and Campo Pisano mines [5,34]. The deep layers showed acidic pH values due to the pyritic tailings rich in pyrite and with low carbonate content [35]. Indeed, the oxidation of pyrite generates H_2_SO_4_ and involves a sequence of reactions beginning with the release of Fe^2+^, which is converted into Fe^3+^ under oxidizing conditions. In pH > 4.5, Fe^3+^ precipitates as a hydroxide generating more acidity. On the contrary, in pH < 4.5, the Fe^3+^ can act as an oxidant of the pyrite to generate much greater acidity [54]. In this regard, the oxidation of sulfur generates sulfate anions that form soluble salts [55], and release large amounts of iron [56]. Unlike the soil in deep layers, the soil around the root samples was mainly characterized by a pH close to neutral and more total carbon and carbonate contents (Table 1). The neutral pH (6.8–7.2) and higher organic carbon content in the CP uppermost soil can decrease the availability of metals (Zn, Pb and Cd) and enhance plant growth to facilitate the phytostabilization process [57,58,59]. Moreover, other studies have also suggested that soil organic amendments can decrease metal availability by the formation of organic metallic complexes [60,61]. According to García-Carmona et al. (2019a) and García et al. (2009), pH appears to be the main property controlling Zn and Cd availability, increasing its solubility in acidic soil conditions [62,63]. However, Jacquat et al. (2008) reported that in neutral soils, organic matter, clay, minerals and carbonates become more influential than pH in Zn availability [64]. Moreover, Sierra-Aragón et al. (2019) reported that Cd availability is strongly reduced by the increase in OC and the rise in pH that causes a decrease in the soluble forms of Cd [65] and the exchangeable fraction of Cd [66,67].

### 4.2. The BCR Fractions Related to the Soil Properties and Mineralogy

The soil around the roots with high total metal content (i.e., CP2) (Figure 3) showed a higher ratio of metals extracted in F1 (Figure 4). The Pearson analysis showed highly significant correlations between the total Zn, Pb and Cd content and all metals were found in the F1 fraction (Table 2) which indicates that the metal availability increased with the total metal content. This is in agreement with the previous studies carried out by Rodríguez et al. (2009), Fernández-Ondoño et al. (2017) and Swed et al. (2022) [11,21,68].

#### 4.2.1. Metals in the Exchangeable Fraction (F1)

The results showed that Cd was mainly associated with the exchangeable fraction (F1) more than Zn and Pb in all contaminated soil samples except S1-3 (Figure 4). Similar results were obtained by Favas et al. (2011) in the Ervedosa mine area of northeastern Portugal, and Swed et al. (2022) in the Silesia-Cracow region in southern Poland [68,69]. This may be due to (i) the presence of carbonates and alkaline waste materials originated from the Monteponi area (SW-Sardinia) that have been loaded on the surface materials of CP mine waste; (ii) in the soil around the root samples, the presence of sulfide and sulfate (Figure 2) and the sulfuric acid production caused by sulfide weathering enhanced the high content of Cd that was found in F1 fraction; (iii) the findings of Jerzykowska et al. (2014) and Sutley et al. (1999) explained that Cd is mainly associated with smithsonite (ZnCO_3_) in calamine mine areas [70,71]. This could describe the highest exchangeable fraction (F1) of Cd (72%) and also Zn (51.8%) measured in the deep soil layer of the S2-2 sample (Figure 4), where smithsonite and Zn-bearing dolomite were detected as the most important host minerals of Cd. It should be noted that the highest total Cd and Zn content was measured in the same soil sample.

The results showed that Zn and Cd contents in all BCR fractions were highly positively correlated with their concentration in DTPA-exchangeable availability in the soil around the root samples (Table 2). This suggests that these metals may be controlled by the rhizosphere processes. Further, the results of the bioavailable Zn and Cd contents in the CP soil around the root samples indicated that the extraction in DTPA was weaker than the dilute acetic acid applied in the exchangeable fraction of BCR (F1). Indeed, Zn and Cd were more bioavailable during the first BCR fraction (up to 31.7% for Zn and 39.5% for Cd) (Figure 4), compared to DTPA-exchangeable fraction (up to 7.2% for Zn and 13.8% for Cd) (Figure 3). This is in accordance with the findings of Luo et al. (2019) that investigated the phytostabilization of Zn and Cd in a smelting slag site in northwestern Guizhou, China [59].

Moreover, the F1 fraction showed the lowest Zn and Pb which were found in the deep soil samples of amended plot S1-3 and S3-3. This can be explained by the low total metal and/or carbonate contents that contributed to precipitation of these metals in mineral forms associated with carbonate (F1) [72]. Soil carbonates may affect metal solubility in water through their surface interactions, providing specific adsorption or precipitation reactions [62,73]. However, soil carbonates have a limited capacity of controlling metal water solubilization and severe metal soil pollution can exceed the thresholds of toxicological concern even in carbonated soils [64]. The significant lower content of Pb that was presented in F1 revealed that Pb was less soluble and less mobilized in all the CP-amended soil samples. This can reduce its toxic impact on the environment [74].

#### 4.2.2. Metals in Reducible Fraction (F2)

The results showed a low content of Zn < Cd in the reducible fraction (F2) for CP soil samples. This mainly refers to the presence of Fe-oxides (Figure 2). Moreover, the highest reducible fraction (F2) for Zn (11.5%) and Cd (24.3%) occurred in the soil around the root sample CP1. The Zn-bearing iron oxide phase can be considered to be the main source of Zn minerals mainly detected in the CP uppermost soils and the soil around the root samples (Figure 2). In non-contaminated soils, the elevated concentration of Pb found in F2 that was bound to Fe and Mn oxides (up to 66.8% in B3) could be caused by the adsorption and accumulation of these elements in the oxide form, favored by alkaline conditions (pH = 8.7) that are favorable for the formation of Fe and Mn oxides.

#### 4.2.3. Metals in Oxidizable Fraction (F3)

In CP soil samples, some part of Zn was presented in the oxidizable fraction F3 > F2 (Figure 4). This could be connected to organic material and sulfides which were oxidized to sulfate. Thus, sulfide could partially dissolve and increase the metal content in the soil with a high content of sulfide minerals (i.e., pyrite, sphalerite and galena) [68,75]. In addition, organic matter is a natural sink of Zn in soils where it is easily absorbed [76,77]. Moreover, in the soil around the roots as well as the uppermost soil samples, Pb was partly found in the oxidizable fraction (F3) (Figure 4). According to the findings of Cappuyns et al. (2007) some of the jarosite group minerals could release Pb in the solution after the second step of BCR extraction [48]. This could describe the presence of Pb in the oxidizable fraction (F3). On the other hand, Romero-Freire et al. (2015) and Coppola et al. (2010) pointed out that soil organic matter is one of the main soil properties controlling lead availability, and it can be attributed to the formation of organic complexes [78,79]. Different authors have observed that high levels of organic matter mainly found in the surface layers of soils are an important sink for lead [65,80].

#### 4.2.4. Metals in Residual Fraction (F4)

In the CP soil samples, Zn and Pb were mainly associated with the residual fraction (F4). The highest Zn (up to 87.2%) and Pb (up to 96.2%) were measured in the deep soil layer of compost-amended plot S1-3 (Figure 4). This could be linked to the more acidic soils that indicate metal sulfides and specifically pyrite (Table 1) and the presence of Pb and Zn in minerals with low solubility, such as hemimorphite, smithsonite, anglesite or cerussite even if these minerals could be formally attributed to the first three fractions. The study carried out by Cappuyns et al. (2007) indicated that Zn- sulfide minerals and pyrite may not completely be dissolved in the F3 fraction by H_2_O_2_ and its dissolution can be completed in the last step of BCR fraction [48]. This can also explain the high Zn content in the residual fraction (F4) (Figure 4).

## 5. Conclusions

The BCR results revealed that the uppermost soil and the deep soil layers of mine waste tailing affected by the rich carbonates lithology and mineralogy were significantly different in relation to the soil properties. Moreover, the (Zn, Pb and Cd) bioavailability of metals in the first fraction of BCR was positively correlated with the total metal contents in the soil around the root samples. Cadmium showed the highest bioavailability in the contaminated soil samples as it was found through the first step of BCR fractions (F1).

Zinc was present in F1 as smithsonite (ZnCO_3_) > in F3 as sphalerite (ZnS) > in F2 as Fe-oxide in the soil around the root samples. However, the results showed that Pb and Zn were often found in high percentages in the residual fraction (F4), with the highest percentage measured in deep layers of the compost-amended soil in mine tailing. That was mainly due to the presence of Zn and Pb ore minerals that, having a lower solubility, can resist leaching in F1, F2 and F3 extraction. The different metal fractions indicated that Pb and Zn were less bioavailable for *P. halepensis* roots. It is not clear if the geochemical fractions of metals and immobilization processes were influenced directly by the pyrite dissolution and weathering process or were induced by the rhizospheres and root activities. Further investigations may provide a better insight of *Pinus halepensis* phytostabilization and its physiological adaptation to better trace the mineralization process on similar contaminated mine tailing sites.

## Figures and Tables

**Table 1:** The physico-chemical properties of the contaminated soil samples in the different soil depths (S1, S2 and S3); the soil around the roots of *Pinus halepensis* (CP1, CP2 and CP3); and the non-contaminated soil around the *P. halepensis* roots (B1, B2 and B3); Number after ± is standard deviation.

Soil	Soil Depth (cm)	Soil Properties						Mean Value (mg kg^−1^)	
		pH	EC (dS m^−1^)	CaCO_3_ (%)	Total Carbon (%)	Total Nitrogen (%)	Organic Carbon (%)	Fe-Oxide	Al-Oxide
Bulk soil in-depth									
S1-1	0–20	6.8	12.05	52.07 ± 0.7	8.9 ± 0.1	0.17 ± 0.01	1.9 ± 0.1	10,565.3 ± 301.3	160.4 ± 6.2
S1-2	20–28	4.5	15.3	4.05 ± 0.1	0.71 ± 0.04	0.010 ± 0.001	0.22 ± 0.01	29,658.3 ± 3026.05	17.2 ± 2.9
S1-3	32–38	2.3	29.3	0.1 ± 0.08	0.36 ± 0.001	0.03 ± 0.01	0.020 ± 0.001	14,920.9 ± 751.9	3.4 ± 1.7
S2-1	0–20	7.3	1.9	59.4 ± 0.4	8.3 ± 0.1	0.04 ± 0.02	0.35 ± 0.02	19,160.8 ± 537.9	189.5 ± 10.3
S2-2	37–41	6.7	14.2	37.5 ± 0.5	5.00 ± 0.05	0.020 ± 0.0003	0.020 ± 0.0004	5909.6 ± 128.6	101.5 ± 18.3
S2-3	47–50	7.6	11.3	47.6 ± 0.7	6.59 ± 0.02	0.002 ± 0.001	0.180 ± 0.003	11,498.5 ± 326.8	37.2 ± 4.9
S3-1	0–20	6.9	10.7	53.6 ± 1.5	8.6 ± 0.2	0.15 ± 0.02	1.5 ± 0.2	12,334.9 ± 99.6	112.9 ± 6.7
S3-2	20–45	7.1	11.2	55.5 ± 0.2	7.62 ± 0.08	0.002 ± 0.001	0.140 ± 0.005	17,411.7 ± 2030.7	61.2 ± 6.3
S3-3	56–66	2.5	19.1	1.40 ± 0.02	0.38 ± 0.005	0.02 ± 0.01	0.190 ± 0.002	42,948.9 ± 5084.9	14.3 ± 3.9
Bulk soil around the roots of *P. halepensis*									
CP1		7.6	8.3	49.7 ± 1.7	9.97 ± 0.05	0.70 ± 0.08	0.35 ± 0.03	9194.4 ± 129.9	211.95 ± 7.08
CP2		7.7	2.2	45.4 ± 1.2	5.7 ± 0.1	0.27 ± 0.02	0.060 ± 0.002	18,265.6 ± 159.4	112.8 ± 9.8
CP3		7.4	8.8	41.03 ± 1.03	8.9 ± 0.2	0.51 ± 0.03	0.33 ± 0.04	10,936.2 ± 269.4	245.6 ± 2.8
B1		6.7	0.3	0.32 ± 0.02	1.2 ± 0.2	0.06 ± 0.01	0.77 ± 0.07	726.8 ± 116.9	215.2 ± 14.3
B2		7.9	3.9	4.07 ± 0.30	10.07 ± 0.04	0.66 ± 0.01	7.61 ± 0.04	992.2 ± 26.8	2323.9 ± 37.9
B3		8.8	1.1	19.03 ± 0.50	3.83 ± 0.08	0.080 ± 0.005	0.78 ± 0.06	467.6 ± 62.5	195.2 ± 8.4

**Table 2:** The correlation matrix between BCR fractions and the total metal content in soil (T-Zn; T-Pb; T-Cd), the total metal content in *Pinus halepensis* root (Zn-R; Pb-R; Cd-R), the bioavailable content of Zn, Pb and Cd (DTPA), the sum of BCR fractions (SF-Zn; SF-Pb; SF-Cd), and the soil characteristics; Correlation (r > 0.8) is significant at the 0.05 level (2-tailed).

Samples	F1			F2			F3			F4		
	Zn	Pb	Cd	Zn	Pb	Cd	Zn	Pb	Cd	Zn	Pb	Cd
Bulk soil around the roots of *P. halepensis*												
T-Zn	0.96	0.86	0.90	0.81	-	-	0.89	0.92	0.93	0.88	-	0.80
T-Pb	0.98	0.92	0.94	0.76	0.85	-	0.86	0.95	0.97	0.89	0.83	-
T-Cd	0.92	0.95	0.96	-	0.92	-	-	0.96	0.94	0.87	0.87	-
Zn-R	0.96	0.98	.99	-	0.95	-	-	0.98	0.99	0.87	0.90	-
Pb-R	0.94	0.99	0.99	-	0.97	-	-	0.97	0.97	0.86	0.93	-
Cd-R	0.93	-	0.82	0.9	-	-	0.98	0.84	0.88	0.80	-	-
DTPA-Zn	0.83	-	0.88	0.80	-	0.97	0.88	-	0.92	0.85	0.82	0.89
DTPA-Pb	0.82	0.83	-	0.85	0.87	-	-	0.81	-	-	-	-
DTPA-Cd	0.85	-	0.89	0.80	-	0.98	0.89	-	0.93	0.87	0.84	0.90
SF-Zn	0.99	-	0.98	0.86	-	0.95	0.98	0.97	0.95	0.96	0.96	0.98
SF-Pb	0.96	0.91	0.95	0.73	0.92	0.82	0.87	0.99	0.92	0.94	0.98	0.89
SF-Cd	0.98	0.92	0.99	0.85	-	0.98	0.97	0.94	0.99	0.98	0.96	0.98
pH	0.86	-	-	-	-	-	-	0.84	0.82	0.89	-	0.94
EC	-	-	-	-	-	-	-	-	-	-	-	-
CaCO_3_	0.88	-	0.80	0.92	-	0.98	0.92	0.80	0.94	0.88	0.83	0.94
Total C	-	-	0.92	-	-	-	-	-	-	-	-	-
Total N	-	-	-	-	-	-	-	-	-	-	-	-
OC	-	-	-	-	-	-	-	-	-	-	-	-
Fe-oxide	0.97	0.80	0.98	0.74	0.82	0.90	0.93	0.98	0.98	0.99	0.94	-
Al-oxide	-	-	-	-	-	-	-	-	-	-	-	-
Bulk soil in-depth												
T-Zn	0.85	-	0.96	-	-	-	-	-	-	0.89	-	0.90
T-Pb	-	-	-	-	-	-	-	-	-	-	-	-
T-Cd	0.86	-	0.93	-	-	-	-	-	-	0.89	-	0.94
SF-Zn	0.97	0.97	.95	0.81	-	-	-	-	-	0.89	-	0.91
SF-Pb	-	-	-	-	-	-	-	-	-	-	0.80	-
SF-Cd	0.90	-	0.97	0.80	-	-	-	-	-	0.88	-	0.93
pH	-	-	-	0.85	-	-	0.89	-	-	-	-	-
EC	-	-	-	-	-	-	-	-	-	-	-	-
CaCO_3_	-	-	-	0.92	-	0.85	0.89	0.83	0.89	-	-	-
Total C	-	-	-	0.91	-	0.88	0.87	0.85	0.90	-	-	-
Total N	-	-	-	-	-	-	-	-	-	-	-	-
OC	-	-	-	-	-	-	-	-	-	-	-	-
Fe-oxide	-	-	-	-	-	-	-	-	-	-	-	-
Al-oxide	-	-	-	-	-	0.94	-	0.92	0.92	-	-	-

## Data Availability

Not applicable.

## Supplementary Materials

The following supporting information can be downloaded at: https://www.mdpi.com/article/10.3390/toxics10120728/s1, **Table SM1:** Soil sampling sites, location and the number of collected samples; **Figure SM2:** Soil samples selected from the different depth layers of core drilled samples in Campo Pisano mine tailing; **Figure SM3**: XRD patterns of *Pinus halepensis* root and CP polluted soil; **Figure SM4**: Spectroscopy-scanning Electron Microscopy (SEM) analysis on the soil around the roots and on the root surface of *Pinus halepensis*; **Figure SM5**: Biological concentration factor (BCF) calculated for all substrates collected around the roots of *Pinus halepensis;* **Figure SM6:** Metal concentration in the roots of *Pinus halepensis* samples; **Table SM7**: The ratio of metal concentrations extracted in the BCR fractions; **Figure SM8**: PCA for the collected soil around the *Pinus halepensis* root samples.
