# Supplementary material for: Pinus halepensis in Contaminated Mining Sites: Study of the Transfer of Metals in the Plant–Soil System Using the BCR Procedure
Source: Toxics. 2022 Nov 26;10(12):728. doi: 10.3390/toxics10120728 (PMC9785362; doi:10.3390/toxics10120728)
Supplement: Supplementary file 1 [file toxics-10-00728-s001.zip › toxics-1985642-supplementary.pdf]

toxics

MDPI

Supplementary Materials: *Pinus halepensis* in Contaminated Mining Sites: Study of the Transfer of Metals in the Plant–Soil System Using the BCR Procedure

Pegah Kharazian, Emilia Fernández-Ondoño, Maria Noelia Jiménez, Manuel Sierra Aragón, Antonio Aguirre-Arcos, Gianluigi Bacchetta, Giovanna Cappai and Giovanni De Giudici

Table SM1. ***Supplementary Materials.*** Soil sampling sites, location and the number of collected samples; **SR**: soil around root, **SC**: soil core samples in-depth, **R**: root samples

Sampling points	Distance from *P. halepensis* in the plot	Code	Soil depth (cm)	nº of samples		
				SR	SC	R
In the CP amended plot	--	CP1		1	-	1
Out of CP amended plot	3–4 m	CP2		1	-	1
Out of CP amended plot	6–7 m	CP3		1	-	1
Not contaminated site (Santa Margherita)	20 cm	B1		1	-	-
Not contaminated site (Calamosca)	20 cm	B2		1	-	-
Less contaminated site (Fontanamare)	20 cm	B3		1	-	-
In the CP amended plot	50 cm	S1-1	0–20	-	1	-
		S1-2	20–28	-	1	-
		S1-3	32–38	-	1	-
Out of CP amended plot	4–5 m	S2-1	0–20	-	1	-
		S2-2	37–41	-	1	-
		S2-3	47–50	-	1	-
In the CP amended plot	1–2 m	S3-1	0–20	-	1	-
		S3-2	20–45	-	1	-
		S3-3	56–66	-	1	-

Figure SM2. ***Supplementary Materials.*** Soil samples selected from the different depth layers of core drilled samples in Campo Pisano mine tailing; numbers (1, 2 and 3) indicate the three selected soils for each core sample.

S1 **S2** **S3**

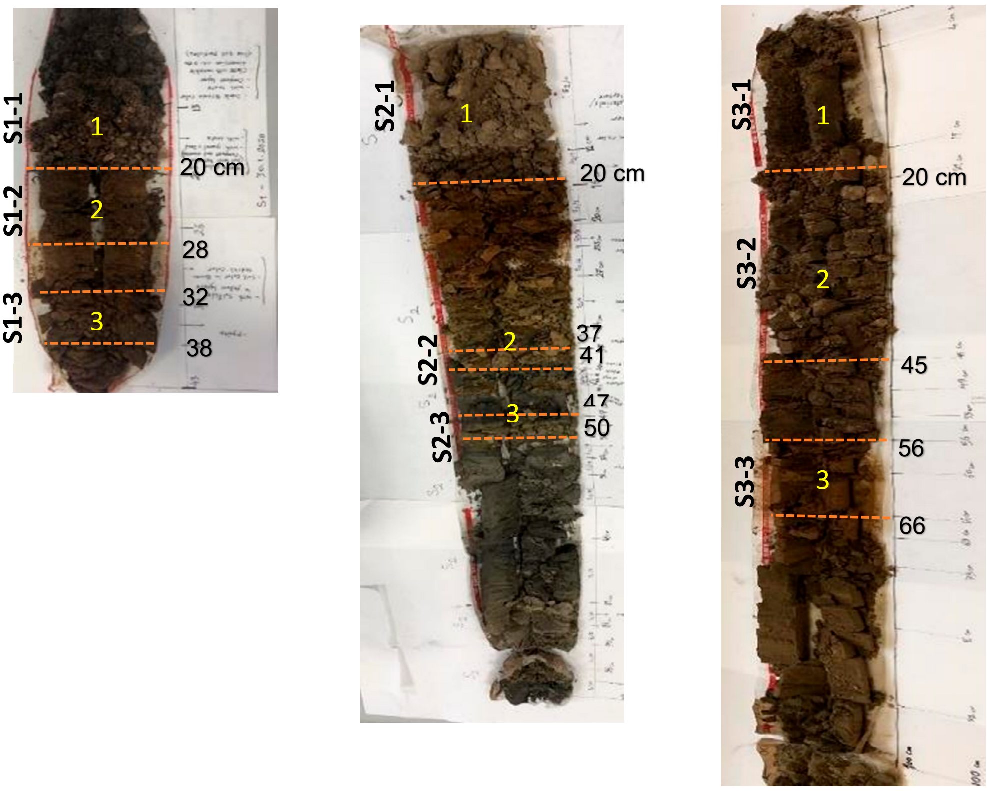

Figure SM3. ***Supplementary Materials.*** XRD patterns of *Pinus halepensis* root and CP polluted soil samples; Mineral legend: Gypsum (G), Quartz (Q), Dolomite (D), Pyrite (P), Muscovite (M), Silica (S), Whewellite (W), Smithsonite (Sm)

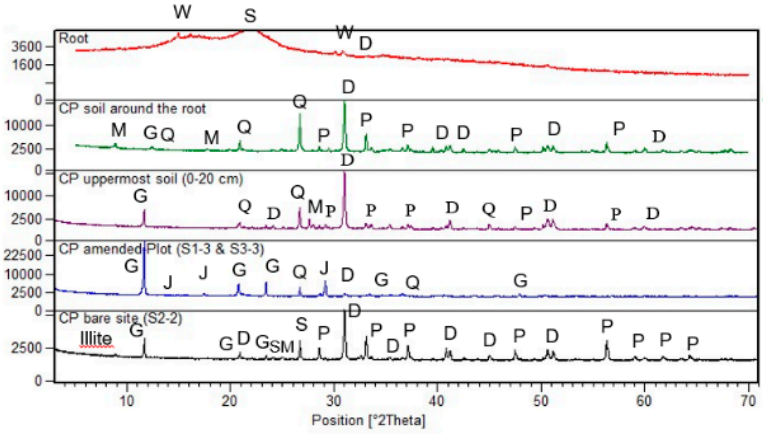

Figure SM4. ***Supplementary Materials.*** Spectroscopy-scanning Electron Microscopy (SEM) analysis on the soil around the roots (a,b and c) and on the root surface (d, e and f) of *Pinus halepensis* grows in Campo Pisano contaminated mine site (CP1, CP2 and CP3); The indicated numbers (1–11 for soil and 1–9 for roots samples) are the points where EDS spectra were acquired.

Soil

CP1

CP2

CP3

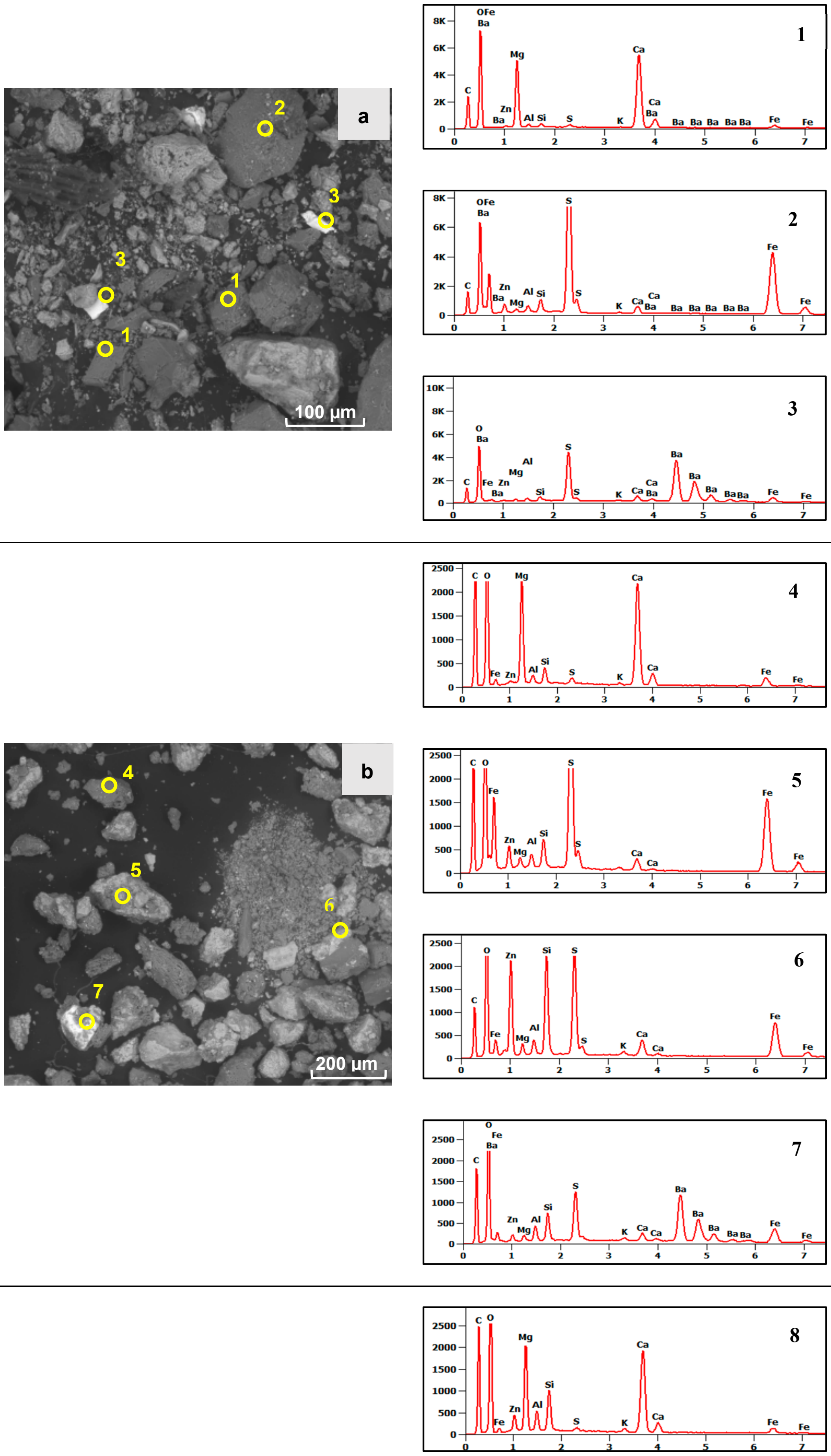

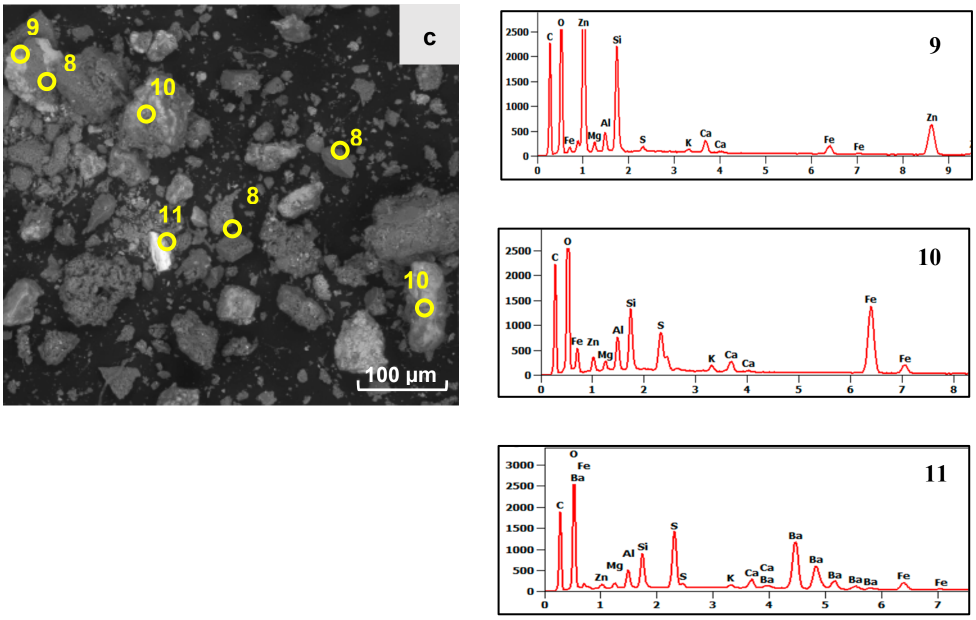

Roots

CP1

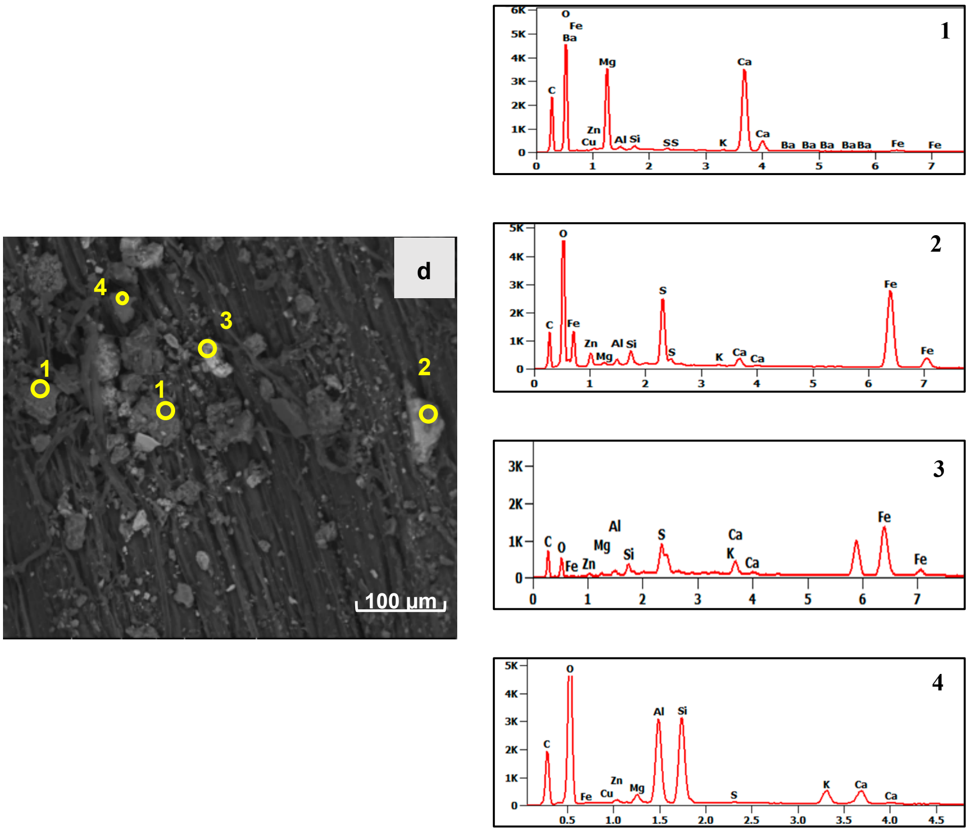

CP2

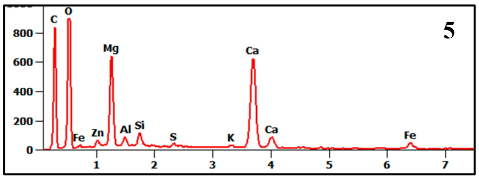

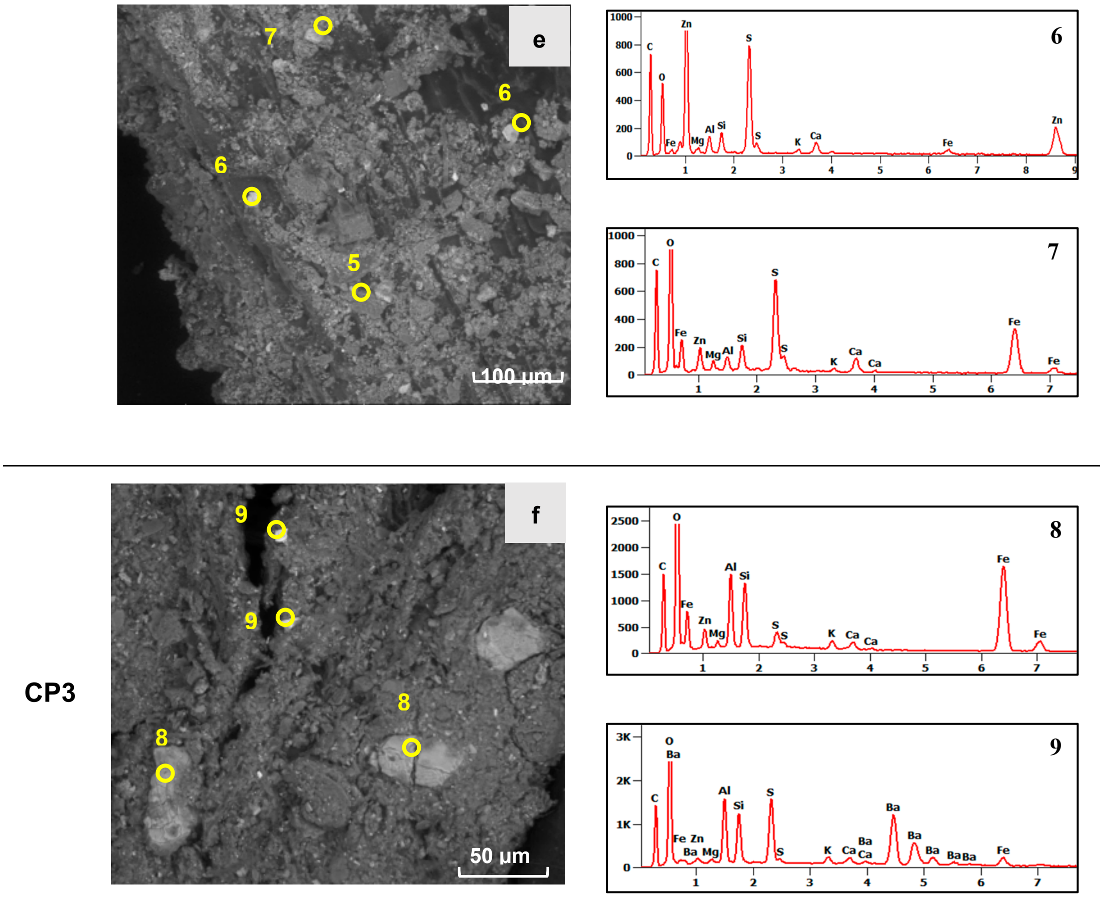

Figure SM5. ***Supplementary Materials.*** Biological concentration factor (BCF) calculated for all substrates collected around the roots of *Pinus halepensis* [39].

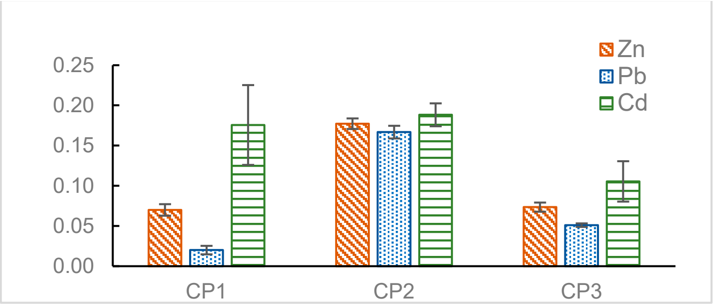

Figure SM6. ***Supplementary Materials.*** Metal concentration in the roots of *P. halepensis* samples exploited from Kharazian et al (2022) [39]

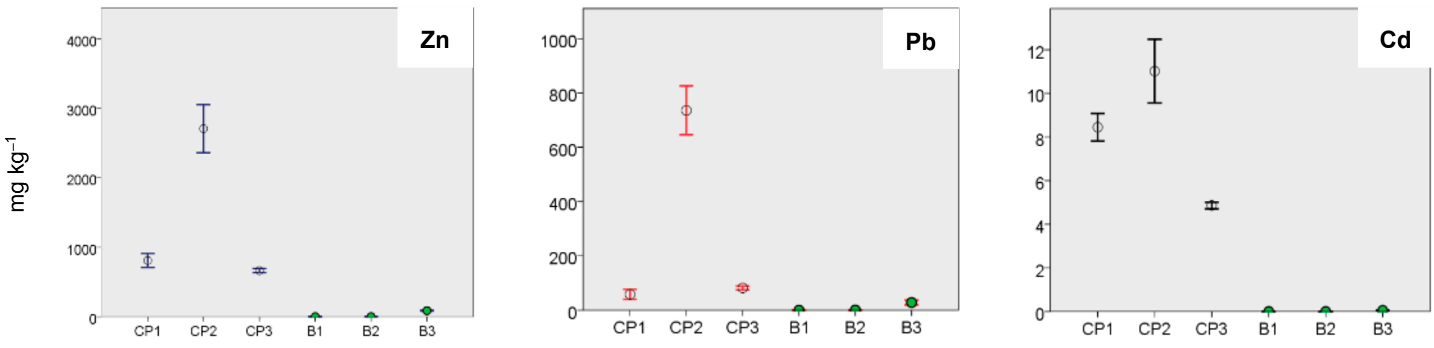

Table SM7. ***Supplementary Materials***. The ratio of metal concentrations extracted in the BCR fractions (F1, F2, F3 and residue F4) in all collected soil samples

Soil depth (cm)		Zn (%)				Pb (%)				Cd (%)			
		F1	F2	F3	F4	F1	F2	F3	F4	F1	F2	F3	F4
**Soil in-depth samples**													
S1-1	0–20	27.2	8.2	13.9	50.3	5.8	12.2	37.3	44.7	44.05	20.8	16	19.2
S1-2	20–28	9.8	2.6	17.7	69.9	0.8	13.8	0.2	85.2	31.7	10.9	28	29.3
S1-3	32–38	3.3	0.1	9.3	87.2	0.1	3.5	0.2	96.2	14.7	6	28.5	50.8
S2-1	0–20	30.3	8.9	14.9	45.8	12.3	10.1	27.5	50.1	55.2	18.7	15	11.1
S2-2	37–41	51.8	4.9	7.9	35.3	29.8	22.3	4.8	43.1	72	7.3	6.8	13.9
S2-3	47–50	26.1	6.1	22.1	45.7	26.1	10.9	12.7	50.3	60.9	7.3	12.9	18.9
S3-1	0–20	34	11	20.1	34.8	2.7	5.6	22.8	68.9	45.4	22.3	17.9	14.3
S3-2	20–45	45.3	9.5	13.2	31.9	4.3	5.2	20.8	69.6	59.1	14.8	13.3	12.8
S3-3	56–66	10.8	1.2	10.2	77.8	0.5	5.1	0.3	94.1	50.4	9.8	16.3	23.4
**Soil around the roots of *P. halepensis***													
CP1		28.2	11.5	19.2	41.2	2.3	5.5	30.5	61.7	32.6	24.3	18.6	24.5
CP2		31.7	8.3	15.4	44.6	20.1	13	22.5	44.7	39.5	18.5	19	23
CP3		28.1	9.9	14.6	47.4	4.3	10.9	20.2	64.7	35.9	23.7	19.2	21.2
B1		19.01	13.8	13.3	53.9	0.17	32	0.29	67.5	5.7	7.5	7.5	79.3
B2		3.5	29.5	6.5	60.4	0	53.8	13.3	32.9	11.8	46.8	5	36.4
B3		32.5	57.9	5.1	4.6	21.4	66.8	4.1	7.6	48.9	36.4	10.1	4.6

Figure SM8. ***Supplementary Materials.*** PCA for the collected soil around the *Pinus halepensis* root samples. Elements extracted in F1, F2, F3, F4, BCR fractions; the total metal content in soil (T-Zn; T-Pb; T-Cd); the sum of BCR fractions (SF-Zn; SF-Pb; SF-Cd); electrical conductivity (EC); organic carbon (OC); total nitrogen (T-N); total carbon (T-C), Aluminum oxide (Al OX), iron oxide (Fe OX), bioavailable fractions of metals (B-Zn, B-Pb, B-Cd); in Campo Pisano contaminated and non-contaminated soil around the *P. halepensis* root samples.

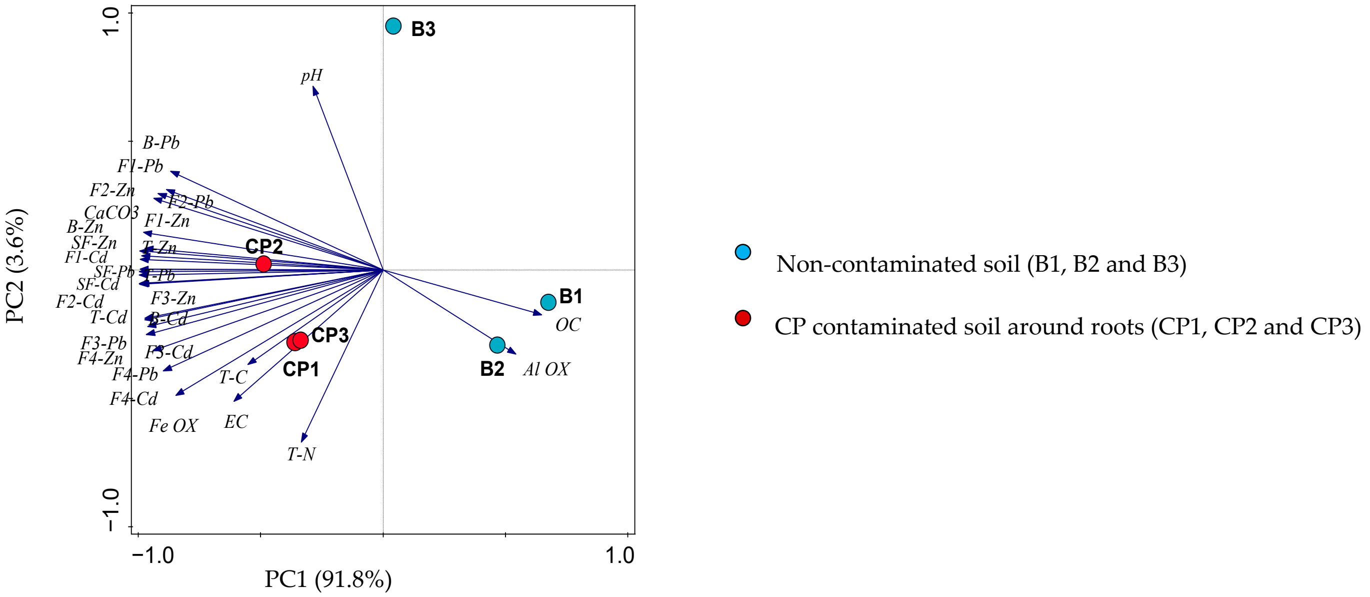

SM8 shows 91.8% of the total variation in the first principal component (PC1) for finding the correlation between the same parameters in the soil samples around the roots. Most of the parameters are mainly presented in the negative side of axis PC1, while only Organic Carbon (OC) and Al-oxide are presented in PC1 with positive correlation. The parameters, such as pH, $CaCO_3$, Fe-oxide and EC as well as Zn, Pb and Cd extracted in all BCR fractions show strongly negative correlation in the inverse section of CP1. The second principal component (PC2) explained 3.6% of the total variation with a high positive correlation for pH and negative correlation for total nitrogen (T-N). The non-contaminated soils (B1 and B2) are distributed in the positive side of the first axis show a positive correlation with OC and Al. Moreover, the soil around the root samples (CP1, CP2 and CP3) are distributed on the negative side of PC1 axis and exhibited high levels of $CaCO_3$, pH, Zn, Pb and Cd extracted in all BCR fractions and the total BCR fractions. Indeed, the availability of Zn, Pb and Cd extracted through BCR fractions are decreased in CP soil around the roots which have high Al and organic carbon.
